# Factors influencing changes in body composition and nutritional status in patients with hyperacute stroke: prospective study

**DOI:** 10.3389/fnut.2025.1548796

**Published:** 2025-03-18

**Authors:** Hiroshi Irisawa, Tomoyuki Nakamura, Yumi Chiba, Mitsuki Hirota, Hajime Hoshiai, Takashi Mizushima

**Affiliations:** Department of Rehabilitation Medicine, Dokkyo Medical University, Mibu, Japan

**Keywords:** malnutrition, stroke, nutritional support, treatment outcome, muscles, prognosis

## Abstract

**Background and aims:**

Muscle loss not only reduce the effectiveness of the recovery period of rehabilitation after stroke but also prolongs the length of hospital stay. Therefore, it is crucial to maintain muscle mass during the hyperacute phase of stroke. We aimed to investigate the factor that influence changes in muscle mass and quality in patients with hyperacute stroke by using a body composition analyzer.

**Methods:**

Body composition assessment was performed on 156 patients admitted to the stroke care unit at the time of admission and 1 week later. Additionally, associations between rehabilitation intervention time, nutritional dosage and administration method, and stroke severity were examined to which factors were affecting body composition.

**Results:**

Muscle mass and quality significantly decreased in both men (SMI: 7.41 ± 1.26 to 7.22 ± 1.23 kg/m^2^, *p* < 0.005, phA: 5.5 ± 1.24 to 5.31 ± 1.29 degree, *p* < 0.005) and women (SMI: 6.04 ± 1.30 to 5.08 ± 1.20 kg/m2, *p* < 0.005, phA: 4.58 ± 0.85 to4.18 ± 0.82 degree, *p* < 0.005)1 week after admission. Rehabilitation intervention time [odds ratio (OR) = 2.12; 95% CI: 1.28–4.47, *p* = 0.01], and high calorie dosage (OR = 1.53; 95% CI: 1.14–3.21, *p* = 0.03) significantly reduced the loss of muscle mass. However, stroke severity did not affect variations in body composition.

**Conclusion:**

Deterioration in muscle mass and quality was observed during the hyperacute phase of stroke. The risk of muscle mass loss may be reduced with appropriate rehabilitation intervention and diet therapy from the early stage of hospitalization. Therefore, providing early rehabilitation intervention and nutritional management in the hospitalization phase are important to improve treatment effectiveness. In hyperacute stroke treatment, rehabilitation and nutritional administration should be provided as early as possible.

**Clinical trial registration:**

https://center6.umin.ac.jp/cgi-open-bin/ctr/ctr_view.cgi?recptno=R000053017, identifier UMIN-CTR UMIN000046467.

## 1 Introduction

The current condition of sarcopenia is widely recognized, and its presence worsens the prognosis of various diseases, including cancer (1–3). In contrast, stroke is one of the leading causes of many motion disorders and deaths worldwide, with an average of 17.9 million deaths from stroke since 2019 (4). Recent developments in thrombolytic and endovascular therapies have reduced the number of deaths in developed countries. However, even survivors have sequelae, causing a high medical cost burden (5). Therefore, rehabilitation intervention is provided to reduce disabilities and facilitate social reintegration. Studies have shown that early rehabilitation intervention after stroke is much more effective in reducing disability, as recommended in the guidelines (6). However, it is important not only to initiate rehabilitation interventions early but also to manage sarcopenia. In fact, previous studies have shown that the presence of sarcopenia during rehabilitation intervention delays functional recovery after stroke (7, 8). Hyperacute stroke patients are inactive because they are forced to rest for treatment or their range of activities is limited. In addition, secondary musculoskeletal degeneration occurs in association with the disruption of central nervous system activity. Thus, elderly patients who are hospitalized after a stroke are more likely to develop sarcopenia. Additionally, our studies have shown that not only muscle mass, as assessed by the presence of sarcopenia, but also muscle quality loss has a significant influence on the effectiveness of poststroke rehabilitation intervention (9, 10). It has become clear that muscle weakness cannot be explained only by the reduced muscle mass (11–13). Reduced muscle mass is attributable to the presence of extracellular fat and extracellular fluid in the skeletal muscle tissue. Ryan et al. (14) measured intramuscular fat mass in stroke patients using computed tomography (CT); they found that the intramuscular fat mass on the paralyzed side increased by approximately 25% compared with that on the nonparalyzed side. Presence of intramuscular fat should be considered during the assessment of skeletal muscle mass in stroke patients. Moreover, a high level of intermuscular fat is liable to reduce muscle strength (15). Therefore, both muscle quality as well as muscle mass should be considered during the assessment of muscle strength. Muscle quality is commonly assessed by CT, MRI, and ultrasound (16); however, phase angle (PhA), measured by body composition monitors, has recently been shown to reflect muscle quality. The European Working Group on Sarcopenia in Older People 2019 consensus statement suggested that PhA can be regarded as an index of overall muscle quality (17). Muscle mass decreases in most patients after stroke (18). However, the factors that contribute to muscle mass loss in the hyperacute phase of stroke remain unclear. Therefore, we measured muscle mass using a body composition analyzer during the hyperacute phase of stroke and investigated some factors that might influence changes in muscle mass. We also examined the factors affecting muscle quality and nutritional status. We hypothesized that during the hyperacute phase of stroke, patients’ muscle mass and quality would decline significantly, but that rehabilitation and nutritional management could reduce these losses.

## 2 Materials and methods

**Figure 1:**
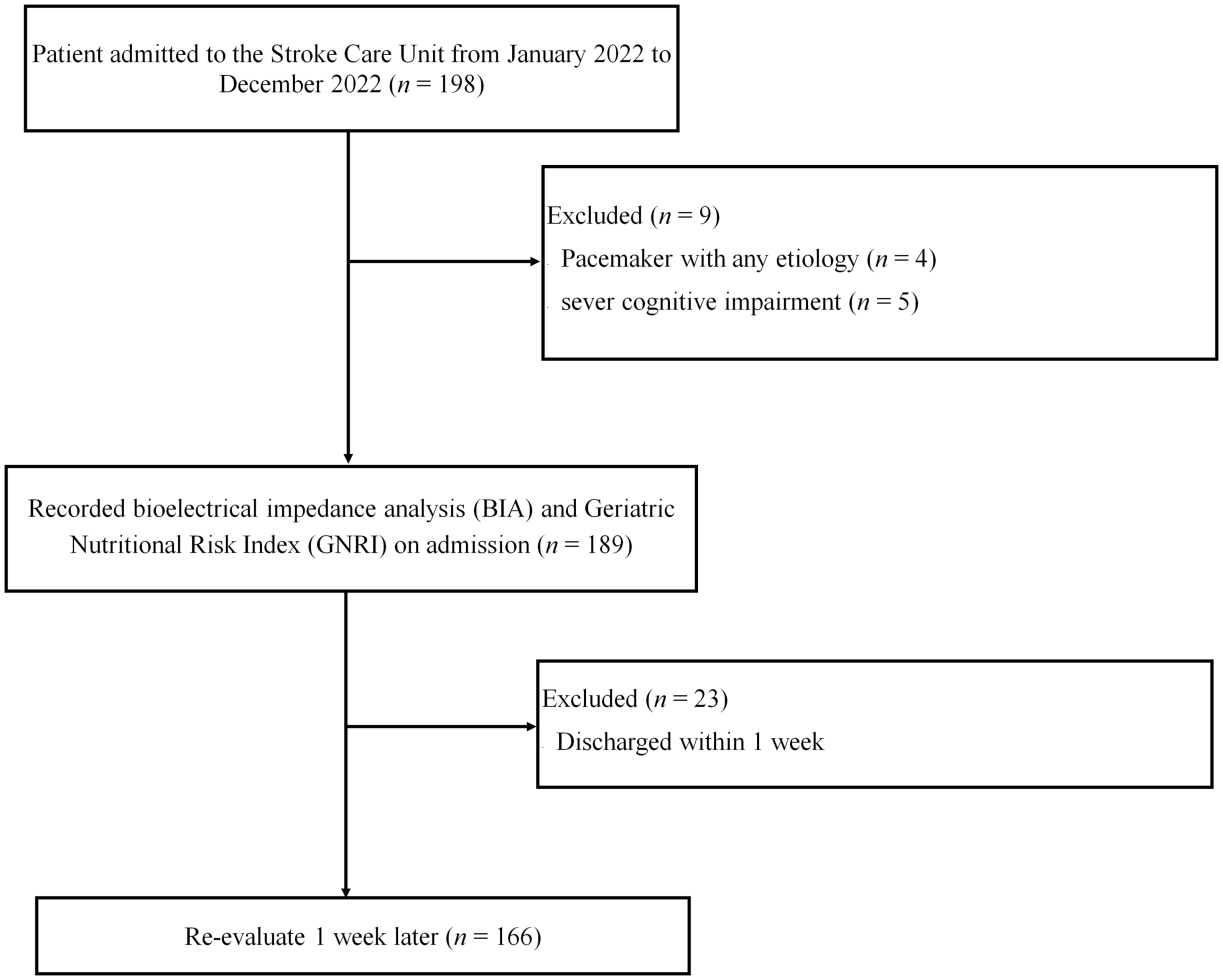
Flowchart outlining the study design as well as the inclusion and exclusion criteria. A total of 198 patients with stroke were admitted to the stroke care unit (SCU); 166 patients (58 women and 108 men) were included in this study. Patients with a pacemaker, severe cognitive impairment, and early discharge were excluded.

We conducted this prospective study at a stroke care unit (SCU) at Dokkyo Medical University Hospital in Japan, in accordance with the Declaration of Helsinki, between January and December 2022. Before the study, all participants signed an informed consent form, and the study protocol was approved by the Ethics Committee of Dokkyo Medical University (R-50-11 J). Additionally, our clinical trial was previously registered in the UMIN-CTR (UMIN000046467). A total of 198 patients with stroke participated in this trial. However, patients with pacemakers and severe cognitive impairment who were discharged early were excluded (Figure 1). Therefore, 166 patients (58 women and 108 men) were included in this study. The National Institutes of Health Stroke Scale (NIHSS) assessed stroke severity at the time of admission of each patient, and posttreatment NIHSS was recorded for patients who underwent thrombolysis or thrombectomy.

### 2.1 Bioelectrical impedance analysis

Body S-10 (InBody Japan, Tokyo, Japan) was used for bioelectrical impedance analysis (BIA), in which a 200-μA current at 5, 50, and 250 kHz was applied after 10 min of rest at air-conditioned room with room temperature adjusted to 25°C and humidity to 60%. BIA was double tested for all patients within 48 h after admission to the SCU and 1 week later. Before the measurements, the patients did not consume food and beverages for 3 h. Noting that all analyses were completed by the same operator. The participants were placed in a supine position where four electrodes were placed on their shaved hands and feet. We used a folding stretcher to measure the patients’ body weight during hospitalization. In addition to the phase angle (PhA) calculated from the impedance values at 50 kHz, the body composition comprising total body water, fat, and skeletal muscle mass was measured using BIA. PhA indicated muscle quality (19). To standardize the values, we used the skeletal muscle mass index (SMI) (20). Additionally, blood albumin levels were measured upon admission. Serum albumin was measured using method of Bromocresol purple (BCP).

### 2.2 Nutritional status

Malnutrition at admission was diagnosed using the Global Leadership Initiative on Malnutrition (GLIM) criteria (21), assessed via two categories, “phenotypic” and “etiologic,” although all the subjects in our study had stroke and were assessed in the “phenotypic” category. GLIM criteria do not describe muscle loss in the Japanese population. Therefore, we used the following two criteria; to assess and diagnose malnutrition: weight loss (>5% in the past 6 months or > 10% beyond 6 months) and low BMI (<18.5 for those <70 years old or < 20.0 for those >70 years old) (22).

To evaluate the nutritional status upon admission to the SCU, we assessed the geriatric nutritional risk index (GNRI), which is commonly adopted as an effective and simple risk index for the prognosis of mortality in hemodialysis and cardiovascular patients (23). The evaluation followed the GNRI formula: GNRI = (1.489 × albumin [g/L]) + (41.7 × [weight/WLo]), where WLo is the ideal body weight (kg) and is computed using the Lorentz equation (for men, H – 100 − [(H − 150)/4]; for women, H – 100 − [(H − 150)/2.5], where H is the height (cm)). In contrast, the daily calorie intake and their weekly average, considering their nutritional administration mode (oral, enteral, or intravenous), were recorded and calculated. Total daily caloric intake was based on the 2020 Dietary Reference Intakes for Japanese, which is defined by the Japanese Ministry of Health, Labor and Welfare (24). The amount of protein given was set at 1.2 g/kg/d. No patients had severe renal dysfunction that necessitated protein intake restriction. Tube and intravenous feeding dosages were similarly determined. In cases of oral intake, supplemental nutritional foods were used to avoid underestimating caloric intake. After 1 week, body composition and nutritional status were compared between the nutritional administration mode (oral, enteral, and intravenous).

### 2.3 Stroke care unit in Japan

The Japanese health insurance system recommends the immediate admission of patients with stroke to a specialized SCU (Suppl 1), where a rehabilitation specialist would develop a therapeutic program including physical, occupational, and speech therapy according to the patient’s medical conditions. We recorded the total number of hours of therapy provided to the patient during the first week of hospitalization. Subsequently, many patients were transferred to the rehabilitation unit for intensive rehabilitation intervention.

### 2.4 Statistical analysis

We used the independent t-test to assess differences between genders. Correlations between lack of malnutrition (GNRI >92), high SMI (men >7.0 kg/m^2^ and women >5.4 kg/m^2^), and high PhA (men >3.5° and women >3.0°) were estimated using odds ratios and 95% confidence intervals obtained from multivariate logistic regression models. Assuming that SMI and PhA vary between men and women, the analysis was conducted on a gender basis (25). The cutoff values of SMI were set according to the criteria of the older Japanese population (26) and the Asian Working Group for Sarcopenia (27). Each variable upon admission was compared between men and women using t-test. Given that 10 items were tested, Bonferroni correction set significance at 0.005. We ensured the normality of each data set using the Shapiro–Wilk method. All data were statistically analyzed using IBM SPSS Statistics version 26 (IBM Corp., Armonk, NY, United States).

## 3 Results

This study included 166 Asian (Japanese) participants: 58 women and 108 men. Table 1 details the characteristics of the subjects. Compared with women, men were older and had higher weight and muscle mass and quality. No significant differences were detected for the other variables. There were no differences between men and women in the percentages of patients with diabetes or hypertension.

**Table 1:** Patients’ characteristics.

	n (M/F)
Subjects	166 (108/58)
Cerebral infarction	125 (85/40)
Cerebral hemorrhage	38 (22/16)
Subarachnoid hemorrhage	3 (1/2)

M, male; F, female.

There were also no patients with high-capacity steroid use, thyroid dysfunction, pituitary tumors, or adrenal tumors that could affect nutrient metabolism. All patients were able to take food orally and were living at home prior to admission. After 1 week, SMI, muscle quality, and nutritional status significantly worsened in both men and women (Table 2). The multivariate analysis showed that rehabilitation intervention time and daily calorie intake were identified as factors influencing muscle mass loss after 1 week, and the nutritional status at the time of admission was considered a factor influencing muscle quality loss after 1 week. Stroke severity and daily calorie intake were also identified as factors influencing the deterioration of nutritional status after 1 week (Tables 3–5; *p* = 0.754, *p* = 0.812, and *p* = 0.689, respectively [Hosmer–Lemeshow test]).

**Table 2:** Subject’s clinical data on admission and 1 week after admission.

	Male	Female	*p*-value
Subjects	108	58	
Age	69.3 ± 15.9	72.4 ± 12.4	0.657
Height (cm)	165.8 ± 7.0	152.0 ± 6.2^*^	0.0012
Weight (kg)	66.8 ± 15.2	54.3 ± 10.8^*^	0.0008
NIHSS	10.2 ± 8.2	9.8 ± 7.9	0.614
Patient with diabetes mellitus	38 (35.2%)	22 (38.3%)	0.528
Patient with hypertension	71 (65.8%)	39 (67.2%)	0.471
On admission			
SMI (kg/m^2^)	7.41 ± 1.26	6.04 ± 1.30^*^	0.0012
PhA (degree)	5.5 ± 1.24	4.58 ± 0.85^*^	0.0031
GNRI	105.7 ± 10.6	108.4 ± 11.0	0.714
Malnutrition	18 (16.7%)	7 (12.0%)	0.349
1 week post admission			
SMI (kg/m^2^)	7.22 ± 1.23^†^	5.08 ± 1.20^*†^	0.0011 (male/female)
			0.0001 (male; on admission)
			0.0001 (female; on admission)
PhA (degree)	5.31 ± 1.29^†^	4.18 ± 0.82^*†^	0.0029 (male/female)
			0.0002 (male; on admission)
			0.0001 (female; on admission)
GNRI	102.8 ± 10.8^†^	104.5 ± 11.1^†^	0.249 (male/ female)
			0.0001 (male; on admission)
			0.0001 (female; on admission)

^*^p < 0.005 vs. male; ^†^*p* < 0.005 vs. on admission.

NIHSS, National Institutes of Health Stroke Scale; SMI, skeletal muscle mass index; PhA, phase angle; GNRI, geriatric nutritional risk index.

**Table 3:** Factors affecting the loss of muscle mass.

	Odds ratio	95% CI	*p*
Rehabilitation intervention time	2.12	1.28–4.47	0.01*
Low NIHSS	0.68	0.24–1.48	0.11
High GNRI	1.61	0.89–3.78	0.17
High calorie intake	1.53	1.14–3.21	0.03*

^*^p < 0.005.

CI, confidence interval; NIHSS, National Institutes of Health Stroke Scale; GNRI, geriatric nutritional risk index.

**Table 4:** Factors affecting the decrease in muscle quality.

	Odds ratio	95% CI	*p*
Rehabilitation intervention time	2.68	0.89–3.47	0.19
Low NIHSS	0.79	0.48–2.17	0.38
High GNRI	1.23	1.07–3.49	0.02*
High calorie dosing	1.36	0.91–3.59	0.15

^*^p < 0.05.

CI, confidence interval; NIHSS, National Institutes of Health Stroke Scale; GNRI, geriatric nutritional risk index.

**Table 5:** Factors affecting the decrease in GNRI.

	Odds ratio	95% CI	*p*
Rehabilitation intervention time	1.97	0.91–4.25	0.16
Low NIHSS	4.51	1.24–8.19	0.03*
High GNRI	1.45	1.07–3.49	0.23
High calorie dosing	6.53	2.68–15.47	0.01*

^*^p < 0.05.

CI, confidence interval; NIHSS, National Institutes of Health Stroke Scale; GNRI, geriatric nutritional risk index.

**Figure 2:**
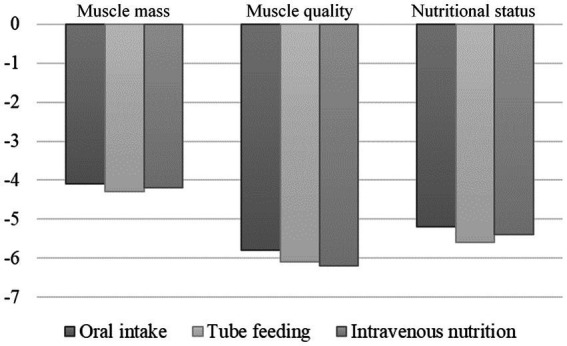
Changes in muscle mass, muscle quality, and nutritional status 1 week after stroke according to the nutritional management method. There were no differences in muscle mass, muscle quality, and nutritional status 1 week after admission when comparing each method for feeding ≥1,200 kcal/day.

When comparing each nutritional administration mode over 1,200 kcal/day, no significant difference was detected in either muscle mass, muscle quality, or nutritional status after 1 week (Figure 2).

## 4 Discussion

Our study revealed that muscle mass, muscle quality, and nutritional status were significantly reduced during the hyperacute phase of stroke, regardless of gender. Patients with less loss of muscle mass had longer rehabilitation and higher nutritional intake. Patients with less loss of muscle quality had better nutritional status before stroke onset. Patients with less deterioration in nutritional status had less severe strokes and higher nutritional doses. To date, none of these reports have examined changes in body composition and nutritional status during the hyperacute phase of stroke.

### 4.1 Relationship between stroke, rehabilitation, and nutrition

Economic losses due to stroke are caused by not only the disease itself but also the loss of activities of daily living (ADL) resulting from poststroke disability. Therefore, to minimize the effects of poststroke disability, rehabilitation intervention should be provided as soon as possible (6). Previous studies have also evaluated the effectiveness of early and intensive rehabilitation interventions (6, 28). However, some patients may struggle to benefit from rehabilitation intervention, especially those with nutritional disorders (29, 30). Additionally, our studies have shown that the effectiveness of stroke rehabilitation is greatly influenced by nutritional status and body composition, such as muscle mass and quality (9, 10). In contrast, both nutritional status and muscle mass decrease in many patients after stroke (31, 32).

According to previous reports, the prevalence of malnutrition in patients with stroke ranges from 6.1 to 62% (33), which is consistent with our findings. Malnutrition is associated with increased incidence of infections, pressure ulcers, gastrointestinal bleeding, prolonged hospital stays, and high mortality rates during hospitalization. In addition, in patients with recovery-phase stroke, malnutrition impedes ADL improvement (9).

Rehabilitation intervention is effective for functional recovery after stroke. However, to enhance it, it is necessary to prevent any deterioration in nutritional status and muscle mass and quality (34). To date, no studies have reported the measurement of nutritional status and muscle mass and quality during the hyperacute phase of stroke.

### 4.2 Relationship between hyperacute stroke, muscle mass, and muscle quality

In this study, nutritional status and muscle mass and quality were measured immediately after stroke onset and 1 week later during the hyperacute phase. Factors that might influence these changes were examined. The results showed that muscle mass was preserved when both rehabilitation intervention time and nutritional administration were increased, which emphasizes the importance of rehabilitation and nutritional management in the hyperacute phase of stroke, as previously indicated (6, 27). Additionally, a healthier nutritional status before stroke onset may prevent any potential muscle quality loss. However, a longer hospitalization period increases the development of muscle catabolism (35), which also progresses with insufficient protein intake, nutritional disorders, and cachexia (36). Therefore, appropriate rehabilitation and nutritional management are thought to halt these catabolic processes.

Poor muscle quality has been shown to delay functional recovery in stroke rehabilitation (9, 10), which is consistent with the importance of nutritional management before hospitalization. Risk factors for stroke include hypertension, dyslipidemia, diabetes mellitus, and other diseases that directly affect nutritional status (37, 38). These relative disorders are common among the elderly, who are primary stroke candidates (39, 40). Appropriate nutritional guidance for groups with these risk factors is considered crucial for an improved health economic policy, both for stroke prevention and for early reintegration into society. As muscle quality begins to deteriorate with an increase in connective and adipose tissues within the skeletal muscle fibers (41–43), a poor premorbid nutritional status may lead to an early muscle catabolism due to either the rest period caused by the disease itself or myosteatosis, which is an inflammatory response after the disease (44).

Stroke severity and nutritional dosage were identified as factors that may worsen the nutritional status in the hyperacute phase of stroke. It has been reported that oral nutritional intake becomes more difficult with increasing stroke severity (45). Furthermore, the prevalence of dysphagia in stroke is estimated to be up to 80%, which also increases with stroke severity (46). Thus, there is a close relationship between stroke severity and nutritional disorders, which may explain the results seen in this study.

Although the study population consisted of stroke patients, 75% of the patients were ischemic, and the remainder were hemorrhagic. This proportion aligns with the proportion of strokes in Japan (47), indicating no bias in the study population. The NIHSS severity guided our analysis, as stroke symptoms are more closely related to disability extent than stroke type.

### 4.3 Hyperacute stroke and nutritional administration mode

Moreover, in the hyperacute phase of stroke, no significant differences in muscle mass, muscle quality, or nutritional status were found after 1 week of oral, enteral, or intravenous feeding when compared with groups receiving the same amount of diet (≥1,200 kcal/day). However, enteral feeding, which is recommended by ESPEN Guidelines in patients with stroke (48), may be difficult in the hyperacute phase because of repeated vomiting caused by cerebellar symptoms and cerebral hypertension. Enteral nutrition is less likely to cause symptoms such as vomiting, nausea, and discomfort due to increased abdominal pressure, symptoms that make rehabilitation difficult. Therefore, patients who have difficulty with enteral nutrition should not be fasted easily, but should first be treated with intravenous nutrition and then transitioned to enteral nutrition after a thorough evaluation.

Inflammatory cytokines and hormones induced by invasion increase the rate of degradation of endogenous proteins (3 times that of normal), inhibit increased protein catabolism and anabolism, and cause symmetrical limb muscle weakness due to prolonged bed rest (49, 50).

In patients with multiple organ dysfunction, muscle mass is reduced by up to 1 kg/day due to hypercatabolism within 10 days after admission to the ICU (51). To replenish this loss of protein, 1.2 to 2.5 g/kg/day of protein should be administered to promote protein synthesis and maintain muscle mass (52).

### 4.4 Limitations

This study has several limitations. First, the study was conducted in one institution, which may have biased the patients’ demographics. The study was conducted in a central regional medical center, minimizing bias due to patient transport from a regional area with a population of approximately two million. Second, there is a large difference between gender, male to female ratio was approximately 2:1, and the number of cases may not be sufficient, particularly for women. Therefore, we analyzed data using rates of change to consider differences in muscle mass and nutritional status between men and women. Third, the nutritional doses were determined solely on the basis of the total calorie counts without considering the nutritional composition (carbohydrates, fat, protein, salt, sugar, fibers, vitamins, and minerals). In particular, the protein content should have been measured accurately because it directly affects muscle anabolism. Additionally, in oral intake, the real calorie count ingested could be underestimated in the case of leftovers. Fourth, although patients with severe cognitive impairment were excluded in this study, other patients’ preadmission comorbidities, medications, ADLs, and physical functions that could affect the results were not considered. Fifth, the decline in muscle mass and quality observed in this study may have been influenced by the patients’ pre-stroke lifestyle and diet. We were unable to ascertain the patients’ lifestyle prior to hospitalization in this study, and we cannot deny the possibility that this may have influenced the results of this study. Sixth, in this study, the measurement of rehabilitation is focused on time, which complicates assessing the workload, as treatment varies according to patient’s condition. Therefore, the amount of workload applied during the same time of rehabilitation is not similar to patients with minor strokes and those with severe strokes. We recommend considering a method that indirectly measures the amount of workload, via an activity meter instance.

## 5 Conclusion

Muscle mass, muscle quality, and nutritional status were significantly reduced in both men and women within the first week after stroke onset. Patients with shorter rehabilitation time and less nutritional administration had a higher risk of muscle mass loss. Patients with poor nutritional status at admission were at higher risk of muscle quality loss. Patients with higher stroke severity and lower nutritional intake were at higher risk of developing undernutrition. There were no differences in muscle mass loss, muscle quality loss, or worsening nutritional status due to different nutritional administration methods in the hyperacute phase of stroke. In hyperacute stroke, patients who have difficulty with enteral nutrition should not choose fasting, but should instead choose intravenous nutrition. In the hyperacute phase of stroke, it is important to provide nutritional support and rehabilitation to patients as early as possible.

## Data Availability

The raw data supporting the conclusions of this article will be made available by the authors, without undue reservation.

## References

[1] Jiang C Wang Y Fu W Zhang G Feng X Wang X. Association between sarcopenia and prognosis of hepatocellular carcinoma: a systematic review and meta-analysis. Front Nutr. (2022) 9:978110. doi: 10.3389/fnut.2022.978110, PMID: 36590214 PMC9794869

[2] He J Luo W Huang Y Song L Mei Y. Sarcopenia as a prognostic indicator in colorectal cancer: an updated meta-analysis. Front Oncol. (2023) 13:1247341. doi: 10.3389/fonc.2023.1247341, PMID: 37965475 PMC10642225

[3] Park B Bhat S Xia W Barazanchi AWH Frampton C Hill AG. Consensus-defined sarcopenia predicts adverse outcomes after elective abdominal surgery: meta-analysis. BJS Open. (2023) 7:zrad065. doi: 10.1093/bjsopen/zrad065, PMID: 37542472 PMC10404004

[4] World Health Organization. World’s health organization-health topics. (2024). Available online at: https://www.who.int/health-topics/cardiovascular-diseases#tab=tab_1 (Accessed January 28, 2024).

[5] World Heart Federation. World heart report 2023. (2023). Available online at: https://world-heart-federation.org/wp-content/uploads/World-Heart-Report-2023.pdf (Accessed January 28, 2024).

[6] Powers WJ Rabinstein A Ackerson T Adeoye OM Bambakidis NC Becker K. Guidelines for the early management of patients with acute ischemic stroke: 2019 update to the 2018 guidelines for the early management of acute ischemic stroke: A guideline for healthcare professionals from the American Heart Association/American Stroke Association. Stroke. (2019) 50:e344–418. doi: 10.1161/STR.0000000000000211 31662037

[7] Mas MF González J Frontera WR. Stroke and sarcopenia. Curr Phys Med Rehabil Rep. (2020) 8:452–60. doi: 10.1007/s40141-020-00284-2, PMID: 33777503 PMC7990034

[8] Matsushita T Nishioka S Taguchi S Yamanouchi A Okazaki Y Oishi K. Effect of improvement in sarcopenia on functional and discharge outcomes in stroke rehabilitation patients. Nutrients. (2021) 13:2192. doi: 10.3390/nu13072192, PMID: 34202303 PMC8308200

[9] Irisawa H Mizushima T. Correlation of body composition and nutritional status with functional recovery in stroke rehabilitation patients. Nutrients. (2020) 12:1923. doi: 10.3390/nu12071923, PMID: 32610491 PMC7400130

[10] Irisawa H Mizushima T. Assessment of changes in muscle mass, strength, and quality and activities of daily living in elderly stroke patients. Int J Rehabil Res. (2022) 45:161–7. doi: 10.1097/MRR.0000000000000523, PMID: 35170496 PMC9071026

[11] Singer KP Breidahl P. The use of computed tomography in assessing muscle cross-sectional area, and the relationship between cross-sectional area and strength. Aust J Physiother. (1987) 33:75–82. doi: 10.1016/S0004-9514(14)60585-7, PMID: 25025589

[12] Freilich RJ Kirsner RL Byrne E. Isometric strength and thickness relationships in human quadriceps muscle. Neuromuscul Disord. (1995) 5:415–22. doi: 10.1016/0960-8966(94)00078-N, PMID: 7496175

[13] Fukumoto Y Ikezoe T Yamada Y Tsukagoshi R Nakamura M Mori N. Skeletal muscle quality assessed from echo intensity is associated with muscle strength of middle-aged and elderly persons. Eur J Appl Physiol. (2012) 112:1519–25. doi: 10.1007/s00421-011-2099-5, PMID: 21847576

[14] Ryan AS Buscemi A Forrester L Hafer-Macko CE Ivey FM. Atrophy and intramuscular fat in specific muscles of the thigh: associated weakness and hyperinsulinemia in stroke survivors. Neurorehabil Neural Repair. (2011) 25:865–72. doi: 10.1177/1545968311408920, PMID: 21734070 PMC3546168

[15] Akazawa N Harada K Okawa N Tamura K Moriyama H. Muscle mass and intramuscular fat of the quadriceps are related to muscle strength in non-ambulatory chronic stroke survivors: a cross-sectional study. PLoS One. (2018) 13:e0201789. doi: 10.1371/journal.pone.0201789, PMID: 30071100 PMC6072321

[16] Harris-Love MO Avila NA Adams B Zhou J Seamon B Ismail C. The comparative associations of ultrasound and computed tomography estimates of muscle quality with physical performance and metabolic parameters in older men. J Clin Med. (2018) 7:E340. doi: 10.3390/jcm7100340, PMID: 30308959 PMC6210142

[17] Cruz-Jentoft AJ Bahat G Bauer J Boirie Y Bruyère O Cederholm T. Writing Group for the European Working Group on sarcopenia in older people 2 (EWGSOP2), and the extended group for EWGSOP2. Sarcopenia: revised European consensus on definition and diagnosis. Age Ageing. (2019) 48:16–31. doi: 10.1093/ageing/afy169, PMID: 30312372 PMC6322506

[18] Hunnicutt JL Gregory CM. Skeletal muscle changes following stroke: a systematic review and comparison to healthy individuals. Top Stroke Rehabil. (2017) 24:463–71. doi: 10.1080/10749357.2017.1292720, PMID: 28251861 PMC5801663

[19] Akamatsu Y Kusakabe T Arai H Yamamoto Y Nakao K Ikeue K. Phase angle from bioelectrical impedance analysis is a useful indicator of muscle quality. J Cachexia Sarcopenia Muscle. (2022) 13:180–9. doi: 10.1002/jcsm.12860, PMID: 34845859 PMC8818694

[20] Janssen I Heymsfield SB Ross R. Low relative skeletal muscle mass (sarcopenia) in older persons is associated with functional impairment and physical disability. J Am Geriatr Soc. (2002) 50:889–96. doi: 10.1046/j.1532-5415.2002.50216.x, PMID: 12028177

[21] Cederholm T Jensen GL Correia MITD Gonzalez MC Fukushima R Higashiguchi T. GLIM criteria for the diagnosis of malnutrition—a consensus report from the global clinical nutrition community. J Cachexia Sarcopenia Muscle. (2019) 10:207–17. doi: 10.1002/jcsm.12383, PMID: 30920778 PMC6438340

[22] Maeda K Ishida Y Nonogaki T Mori N. Reference body mass index values and the prevalence of malnutrition according to the global leadership initiative on malnutrition criteria. Clin Nutr. (2020) 39:180–4. doi: 10.1016/j.clnu.2019.01.011, PMID: 30712782

[23] Bouillanne O Morineau G Dupont C Coulombel I Vincent JP Nicolis I. Geriatric nutritional risk index: a new index for evaluating at-risk elderly medical patients. Am J Clin Nutr. (2005) 82:777–83. doi: 10.1093/ajcn/82.4.777, PMID: 16210706

[24] Japanese Ministry of Health, Labour and Welfare. Dietary reference intakes for Japanese (2020), Available online at: https://www.mhlw.go.jp/content/10900000/001150922.pdf (Accessed October 2, 2024) (in Japanese).

[25] Janssen I Heymsfield SB Baumgartner RN Ross R. Estimation of skeletal muscle mass by bioelectrical impedance analysis. J Appl Physiol. (2000) 89:465–71. doi: 10.1152/jappl.2000.89.2.465 10926627

[26] Kishimoto M Shide K Tanaka M Wada K Fukuda M Himeno M. A methodological evaluation of body composition analysis for patients with lifestyle-related disorders. Nihon Eiyo Shokuryo Gakkai Shi. (2009) 62:253–8. doi: 10.4327/jsnfs.62.253

[27] Chen LK Woo J Assantachai P Auyeung TW Chou MY Iijima K. Asian working Group for Sarcopenia: 2019 consensus update on sarcopenia diagnosis and treatment. J Am Med Dir Assoc. (2020) 21:300–307.e2. doi: 10.1016/j.jamda.2019.12.012, PMID: 32033882

[28] Sato Y Yoshimura Y Abe T Nagano F Matsumoto A. Hospital-associated sarcopenia and the preventive effect of high energy intake along with intensive rehabilitation in patients with acute stroke. Nutrition. (2023) 116:112181. doi: 10.1016/j.nut.2023.112181, PMID: 37678013

[29] Chen Y Yang X Zhu Y Zhang X Ni J Li Y. Malnutrition defined by geriatric nutritional risk index predicts outcomes in severe stroke patients: a propensity score-matched analysis. Nutrients. (2022) 14:4786. doi: 10.3390/nu14224786, PMID: 36432473 PMC9696179

[30] Qin H Wang A Zuo Y Zhang Y Yang B Wei N. Malnutrition could predict 3-month functional prognosis in mild stroke patients: findings from a Nationwide stroke registry. Curr Neurovasc Res. (2021) 18:489–96. doi: 10.2174/1567202619666211217130221, PMID: 34923942 PMC8972270

[31] Sabbouh T Torbey MT. Malnutrition in stroke patients: risk factors, assessment, and management. Neurocrit Care. (2018) 29:374–84. doi: 10.1007/s12028-017-0436-1, PMID: 28799021 PMC5809242

[32] Li W Yue T Liu Y. New understanding of the pathogenesis and treatment of stroke-related sarcopenia. Biomed Pharmacother. (2020) 131:110721. doi: 10.1016/j.biopha.2020.110721, PMID: 32920517

[33] Foley NC Salter KL Robertson J Teasell RW Woodbury MG. Which reported estimate of the prevalence of malnutrition after stroke is valid? Stroke. (2009) 40:66–74. doi: 10.1161/STROKEAHA.108.518910 19164799

[34] Liu S Liu H Yang L Wang K Chen N Zhang T. A review of rehabilitation benefits of exercise training combined with nutrition supplement for improving protein synthesis and skeletal muscle strength in patients with cerebral stroke. Nutrients. (2022) 14:4995. doi: 10.3390/nu14234995, PMID: 36501025 PMC9740942

[35] Bodine SC. Disuse-induced muscle wasting. Int J Biochem Cell Biol. (2013) 45:2200–8. doi: 10.1016/j.biocel.2013.06.011, PMID: 23800384 PMC3856924

[36] Gordon BS Kelleher AR Kimball SR. Regulation of muscle protein synthesis and the effects of catabolic states. Int J Biochem Cell Biol. (2013) 45:2147–57. doi: 10.1016/j.biocel.2013.05.039, PMID: 23769967 PMC3759561

[37] Barthels D Das H. Current advances in ischemic stroke research and therapies. Biochim Biophys Acta Mol basis Dis. (2020) 1866:165260. doi: 10.1016/j.bbadis.2018.09.012, PMID: 31699365 PMC6981280

[38] Shi Y Guo L Chen Y Xie Q Yan Z Liu Y. Risk factors for ischemic stroke: differences between cerebral small vessel and large artery atherosclerosis aetiologies. Folia Neuropathol. (2021) 59:378–85. doi: 10.5114/fn.2021.112007, PMID: 35114778

[39] Yuan S Larsson SC. Epidemiology of sarcopenia: prevalence, risk factors, and consequences. Metabolism. (2023) 144:155533. doi: 10.1016/j.metabol.2023.155533, PMID: 36907247

[40] Shaw SC Dennison EM Cooper C. Epidemiology of sarcopenia: determinants throughout the lifecourse. Calcif Tissue Int. (2017) 101:229–47. doi: 10.1007/s00223-017-0277-0, PMID: 28421264 PMC5544114

[41] Longo S Coratella G Rampichini S Borrelli M Scurati R Limonta E. Local fat content and muscle quality measured by a new electrical impedance myography device: correlations with ultrasound variables. Eur J Sport Sci. (2021) 21:388–99. doi: 10.1080/17461391.2020.1751306, PMID: 32237960

[42] De Carvalho FG Justice JN Cd FE Kershaw E Sparks LM. Adipose tissue quality in aging: how structural and functional aspects of adipose tissue impact skeletal muscle quality. Nutrients. (2019) 11:2553. doi: 10.3390/nu11112553, PMID: 31652734 PMC6893709

[43] Rahemi H Nigam N Wakeling JM. The effect of intramuscular fat on skeletal muscle mechanics: implications for the elderly and obese. J R Soc Interface. (2015) 12:20150365. doi: 10.1098/rsif.2015.0365, PMID: 26156300 PMC4535407

[44] Li CW Yu K Shyh-Chang N Jiang Z Liu T Ma S. Pathogenesis of sarcopenia and the relationship with fat mass: descriptive review. J Cachexia Sarcopenia Muscle. (2022) 13:781–94. doi: 10.1002/jcsm.12901, PMID: 35106971 PMC8977978

[45] Brandão BC Silva MAOMD Rodrigues CG Damando MD Lourenção LG. Relationship between oral intake and severity of acute stroke. Codas. (2020) 32:e20180154. doi: 10.1590/2317-1782/20202018154, PMID: 33053079

[46] Takizawa C Gemmell E Kenworthy J Speyer R. A systematic review of the prevalence of oropharyngeal dysphagia in stroke, Parkinson's disease, Alzheimer's disease, head injury, and pneumonia. Dysphagia. (2016) 31:434–41. doi: 10.1007/s00455-016-9695-9, PMID: 26970760

[47] Hata J Ninomiya T. Epidemiology of stroke in a general Japanese population: the Hisayama study. J Atheroscler Thromb. (2023) 30:710–9. doi: 10.5551/jat.RV22004, PMID: 37258234 PMC10322733

[48] Thibault R Abbasoglu O Ioannou E Meija L Ottens-Oussoren K Pichard C. ESPEN guideline on hospital nutrition. Clin Nutr. (2021) 40:5684–709. doi: 10.1016/j.clnu.2021.09.039 34742138

[49] Klaude M Mori M Tjäder I Gustafsson T Wernerman J Rooyackers O. Protein metabolism and gene expression in skeletal muscle of critically ill patients with sepsis. Clin Sci. (2012) 122:133–42. doi: 10.1042/CS20110233 21880013

[50] Batt J dos Santos CC Cameron JI Herridge MS. Intensive care unit-acquired weakness: clinical phenotypes and molecular mechanisms. Am J Respir Crit Care Med. (2013) 187:238–46. doi: 10.1164/rccm.201205-0954SO 23204256

[51] Puthucheary ZA Rawal J McPhail M Connolly B Ratnayake G Chan P. Acute skeletal muscle wasting in critical illness. JAMA. (2013) 310:1591–600. doi: 10.1001/jama.2013.278481, PMID: 24108501

[52] Hurt RT McClave SA Martindale RG Ochoa Gautier JB Coss-Bu JA Dickerson RN. Summary points and consensus recommendations from the international protein summit. Nutr Clin Pract. (2017) 32:142S–51S. doi: 10.1177/0884533617693610, PMID: 28388374

